# Attapulgite Nanorods Incorporated MXene Lamellar Membranes for Enhanced Decontamination of Dye Wastewater

**DOI:** 10.3390/nano12183094

**Published:** 2022-09-06

**Authors:** Shiyang Zhang, Zhi Wang, Mingwei Cai, Xiaochuang Lu, Tianju Fan, Ruibin Wang, Yidong Liu, Yonggang Min

**Affiliations:** 1 School of Materials and Energy, Guangdong University of Technology, Guangzhou 510006, China; 2 School of Chemistry and Chemical Engineering, University of South China, Hengyang 421001, China

**Keywords:** MXene, attapulgite, methylene blue, permeation flux, rejection

## Abstract

Due to its unique physical and chemical properties, MXene has recently attracted much attention as a promising candidate for wastewater treatment. However, the low water permeation flux of MXene membrane remains a challenge that has not been fully solved. In this study, attapulgite was used to increase the flux of MXene membrane through a facile one-pot method, during which the MXene nanosheets were self-assembled while being intercalated by the attapulgite nanorods to finally form the composite membranes. Under optimal conditions, an increase of water permeation flux of 97.31% could be observed, which was attributed to the broadened nano-channel upon the adequate intercalation of attapulgite nanorods. Its permeation flux and rejection rate for methylene blue (MB) were further studied for diverse applications. In contrast to bare MXene, the permeation flux increased by 61.72% with a still high rejection rate of 90.67%, owing to the size rejection. Overcoming a key technique barrier, this work successfully improved the water permeability of MXene by inserting attapulgite nanorods, heralding the exciting prospects of MXene-based lamellar membrane in dye wastewater treatment.

## 1. Introduction

MXene is a new class of graphene-analogue two-dimensional materials with abundant oxygen-containing functional groups [1,2]. In terms of structure, MXene is composed of nanosheets that are self-assembled and stacked into membranes under van der Waals forces [3,4]. MXene has narrow interlayer spacing that renders it capable of selectively passing through only small molecule compounds, promising a broad application, especially in the purification of dye pollutants [5,6,7,8]. An excellent rejection rate can be obtained by using MXene membranes; however, a balance between its rejection rate and water permeance is yet to be achieved, which remains a big challenge for its practical application and development [9].

In this case, many efforts have been paid to engineer the interlayer spacing of MXene with various nanomaterials of different appropriate sizes to optimize its pillar effect so as to effectively improve the permeation flux [10,11]. In other words, the insertion of nanomaterials is considered one of the primary choices for the preparation of emerging MXene-based composite lamellar membranes [12] with a well-balanced permeation flux and rejection rate. It was reported by Liu et al. [13] that by incorporating graphene oxide (GO), the interlayer spacing of MXene could be easily tuned with the change of the GO content. In contrast to the reference GO membrane, about a 10-fold increase of pure water permeation flux was reached for the GO-incorporated MXene membrane, with a high rejection above 99.5% towards four commonly used organic dyes. The high water flux was mainly attributed to the moderate increase of the interlayer spacing of the composite membrane and the decrease of oxygen-containing functional groups. By altering the intercalant loading of nano-alumina between MXene nanosheets, their interlayer spacing could also be precisely tailored to enhance the channel stability. Consequently, a high dye rejection was realized under a high permeability of 88.8 L·m^−2^·h^−1^·bar^−1^, which was fourfold that of the unmodified MXene. The separation mechanism was caused by the synergetic effects of the Donnan exclusion effect, size sieving and molecule aggregation of the organics [14]. However, graphene and nano-alumina are expensive and complicated in terms of preparation.

As a naturally occurring clay mineral, attapulgite is widely used as a reinforcement in various composites due to its unique one-dimensional structure, environmental benignancy, hydrophilicity and low cost [15,16]. Its fibrous morphology and abundant surface hydroxyl groups enable it to be well dispersed in water and easily modifiable [17,18]. Hence, attapulgite has already been reported as a good intercalation material of 2D-material membranes to increase their permeability. Zhao et al. [19] successfully fabricated GO/attapulgite composite membranes, in which attapulgite was effectively inserted into GO nanosheets to expand their interlayer spacing. The GO/attapulgite composite membranes showed ≤ seven-fold permeation flux increase compared with that of pure GO membrane, along with a good oil-water separation and oil stain resistance. Wang et al. [20] prepared GO/attapulgite composite membranes by an ultrasonication-assisted physical mixing method. Compared to the pristine GO membrane, the permeation flux of the composite membrane was increased three times, and the rejection rate of the rhodamine B was close to 100%. Dye molecules were efficiently rejected by the synergistic separation mechanism: size exclusion effect and electrostatic interactions. Further investigations indicated that this was attributed to attapulgite that was inserted into the two-dimensional GO membrane, and thus the unimpeded “empty” interlayer spacing or open channels were increased to allow more water molecules to rapidly pass through. However, studies on combining attapulgite with MXene to increase the permeation flux of MXene membrane are currently rare. 

Herein, we developed attapulgite/MXene composite membranes through a facile vacuum-assisted filtration that can be applied in the treatment of dye wastewater. Attapulgite was intercalated into the MXene nanosheets to adjust their interlayer spacing, so as to improve the permeation flux and with a slight decrease in the dye rejection rate. 

## 2. Materials and Methods

### 2.1. Materials

Titanium aluminum carbide (Ti_3_AlC_2_) (particle size < 38 μm) was purchased from Laizhou Kaiene Ceramic Material Co., Ltd. (Laizhou, China). Attapulgite was obtained from Gansu Qianzhan Technology Co., Ltd. (Zhangye, China). Lithium fluoride (LiF) and methylene blue (MB) were supplied by Shanghai alighting biochemical technology Co., Ltd. (Shanghai, China). Dimethyl sulfoxide and concentrated hydrochloric acid were provided by Shanghai Mclean biochemical technology Co., Ltd. (Shanghai, China). and Guangzhou Chemical Reagent Factory (Guangzhou, China), respectively. All reagents used in this research were analytically pure and used without further purification. Cellulose esters microfiltration membrane was provided from Tianjin Jinteng Experimental Equipment Co., Ltd. (Tianjin, China).

### 2.2. Preparation of MXene Nanosheets

MXene was fabricated according to the previous work [11,21]. A total of 3.2 g of LiF were added into 40 mL HCl solution (9 mL·L^−1^) with stirring at room temperature until LiF was dissolved. Then, 2 g of Ti_3_AlC_2_ powder were gradually put into the above mixture solution and stirred continuously at 40 °C for 48 h. After the mixture was washed to neutrality by repeated centrifugation/washing, the precipitate was transferred to a freeze dryer for drying (−60 °C and 22 Pa). The thus-obtained solid (1 g) was put into 20 mL of dimethyl sulfoxide and stirred at 40 °C for 12 h. Subsequently, the mixture was washed/centrifuged (H1850, Hunan Xiangyi Laboratory Instrument Development Co., Ltd., Changsha, China) for 10 min at 8000 rpm several times with deionized water. The precipitate was dispersed into deionized water by ultrasound, followed by centrifugation for 30 min at 3000 rpm. Next, the supernatant including MXene nanosheets was collected for further usage.

### 2.3. Purification of the Attapulgite

Attapulgite was fully ground and sieved using a 400 mesh sieve. A total of 10 g of sieved attapulgite were poured into 400 mL HCl solution with a concentration of 1 mol·L^−^^1^, and stirring was continued until there were no bubbles present in the solution, followed by stirring for 30 min. Finally, the thus-obtained suspension was filtered and thoroughly washed with deionized (DI) water until neutralized (pH of 7). Then, the filter cake was placed in the freeze dryer overnight. Next, all the samples were collected for further usage.

### 2.4. Preparation of MXene/Attapulgite Composite Membranes

**Scheme 1:**
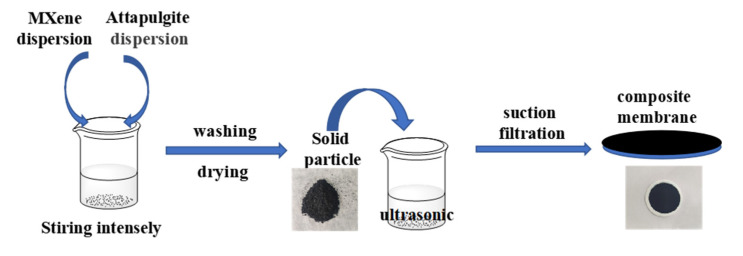

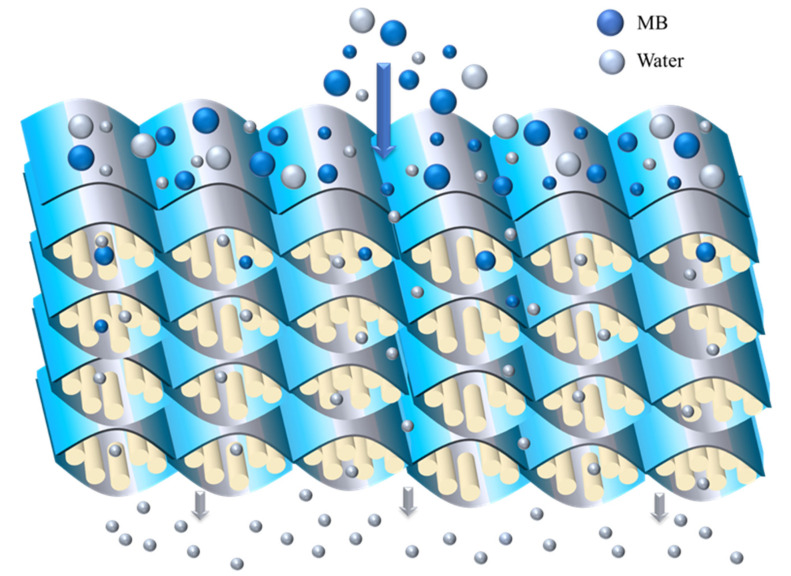
The preparation flow chart and structure diagram of MXene/attapulgite composite membranes.

The preparation method of MXene/attapulgite composite materials was reported in the literature [20]. A total of 0.25 g of pretreated attapulgite were dispersed in 1 L of deionized water, followed by ultrasonication for 30 min to obtain a 0.25 g·L^−1^ uniform attapulgite dispersion. The MXene dispersion was also configured to a concentration of 0.25 g·L^−1^. Next, the MXene were added into the attapulgite dispersion with a certain ratio (MXene: attapulgite = 1:0.25, 1:0.5, 1:1, 1:2, 1:3, and 1:4). Then, the mixed dispersion was vigorously stirred for 6 h to form a homogeneous composite suspension. Finally, the mixed cellulose ester membrane was used as the supporting substrate, and the composite suspension was prepared into a membrane by suction filtration to obtain the MXene/attapulgite composite membranes. The preparation flow chart and structure diagram of MXene/attapulgite composite membranes are shown in Scheme 1.

### 2.5. Characterization

The concentration of MB was measured with a UV-visible spectrophotometer (UV-759CRT, Shanghai Yoke Instrument Co., Ltd., Shanghai, China). The structures of samples were characterized using X-ray diffraction (XRD, Max 2200, Rigaku Co., Ltd., Tokyo, Japan) with CuKα radiation (λ = 1.5406 Å) at a generator voltage of 40 kV. The functional groups of samples were obtained by using a NicoletiS50 Fourier transformed spectrometer (FTIR, Thermo Fisher Scientific, Dreieich, Germany) in the wavenumber range of 400–4000 cm^−1^. The morphology of samples were observed by scanning electronic micrograph (SEM, SU8100, Hitachi Co., Ltd., Tokyo, Japan) at a generator voltage of 15 kV. The internal microstructure of samples was further observed via a transmission electron microscope at a voltage of 200 kV (TEM, JM-2100, JEOL, Tokyo, Japan). The chemical composition was analyzed using X-ray photoelectron spectroscopy (XPS, Escalab 250Xi, Thermo Scientific, Waltham, MA, USA) employing a monochromated Al Kα X-ray source (hν = 1486.6 eV). Water contact angles on the surfaces of MXene and MXene/attapulgite composite membranes were recorded on a Powereach JC2000Cl drop shape analysis system (Shanghai Zhong Chen Digital Technique Equipment Co. Ltd., Shanghai, China). The roughness of the membranes was observed by atomic force microscopy (AFM, Dimension Fast Scan, Bruker, Ettlingen, Germany) using a scanning probe microscope. 

### 2.6. Experimental Method

Both the water flux (the pure water flux and the dye water flux) and dye removal of MXene/attapulgite composite membranes were tested through the terminal filtration device. The filtrate was taken for a volume and concentration test until the performance was stable. The water permeation flux (*F*, L·m^−2^·h^−1^·bar^−1^) and rejection rate (*R*) of membranes were carefully addressed according to a previous work [11]. In detail, *F* and *R* could be calculated according to the following equations:

$$F=\frac{V}{S\times T\times P}$$

where *V* represents the volume (L) of the dye solution that is decontaminated over a period of time, *S* represents the effective membrane area, and *T* and *P* denote the separation time (h) and the operating filtration pressure which is fixed at about 1.0 bar, respectively.

$$R=\frac{C_{0}-C}{C_{0}}\times 100\%$$

where *C*_0_ and *C* represent the dye concentration in the source liquid and the filtrate (mg·L^−1^), respectively.

## 3. Results and Discussion

### 3.1. Morphological, Structural and Chemical Characterization of MXene/Attapulgite Composites

**Figure 1:**
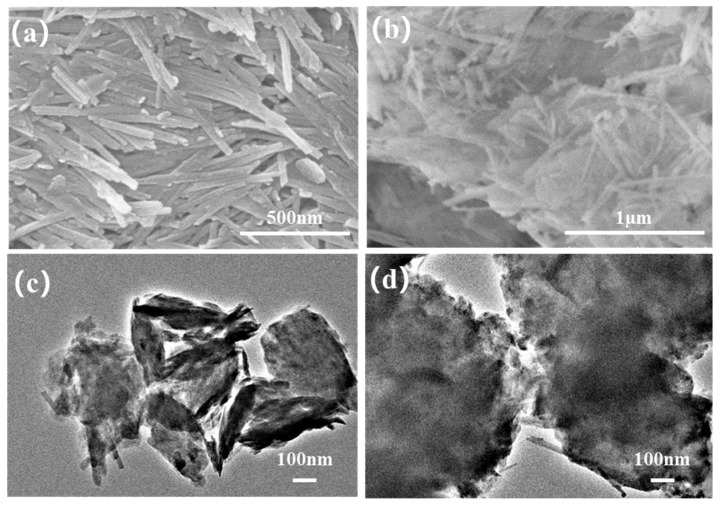
SEM images of (**a**) attapulgite and (**b**) MXene/attapulgite composite (1:2); TEM images of (**c**) attapulgite and (**d**) MXene/attapulgite composite (1:2).

The surface morphologies of attapulgite and the MXene/attapulgite composite were examined by SEM. As depicted in Figure 1a, attapulgite shows a typical nanorod-like structure with a diameter of about 30 nm and a length in the range of 0.5~1 µm, similar to what was reported elsewhere [22]. In the composite, it can be clearly seen from Figure 1b that the rod-shaped attapulgite was loaded on the MXene nanosheets. As further displayed in Figure 1c,d, attapulgite nanorods have a small diameter of about 30~50 nm and are completely wrapped up by MXene, thus creating more nanochannels for the migration of small molecules (like water) [23].

**Figure 2:**
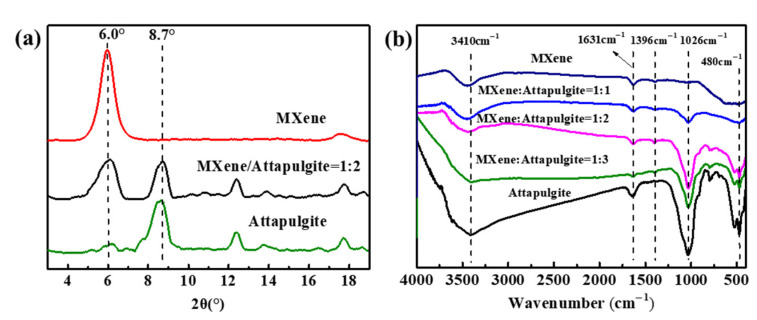
(**a**) XRD results and (**b**) FTIR spectra of attapulgite, MXene and the MXene/attapulgite composite.

Figure 2a exhibits the XRD results of MXene, attapulgite and the MXene/attapulgite composite (1:2). Obviously, MXene presents a strong diffraction peak at 6.0°, which indicates an initial d-spacing of 1.33 nm. For the composite, it is found that its diffraction peaks include a 2θ of 8.7°, which corresponds to the (110) crystal plane of attapulgite [20,24] and the characteristic peak (6.0°) of MXene. Interestingly, the decreased intensity of the latter [25] (easily read from referencing another peak related to MXene around 18°) is evidence of the fact that intercalation might occur between attapulgite and MXene.

The chemical structure and functional groups of the above samples were analyzed by FTIR. As shown in Figure 2b, two characteristic peaks assigned to the stretching vibration of –OH and C=O groups are observed at 3410 and 1631 cm^−1^ for MXene, while the absorption peaks appearing at 3410, 1631, 1026 cm^−1^ and 480 cm^−1^ are attributed to –OH, H_2_O, Si–OH and Si–O groups, respectively [26,27]. To the best of our knowledge, the oxygen-containing groups of MXene and attapulgite are able to interact with each other, which is supported by the gradually weakened oxygen-containing peaks in the FTIR spectra of the composites with an increase of attapulgite loading [28].

**Figure 3:**
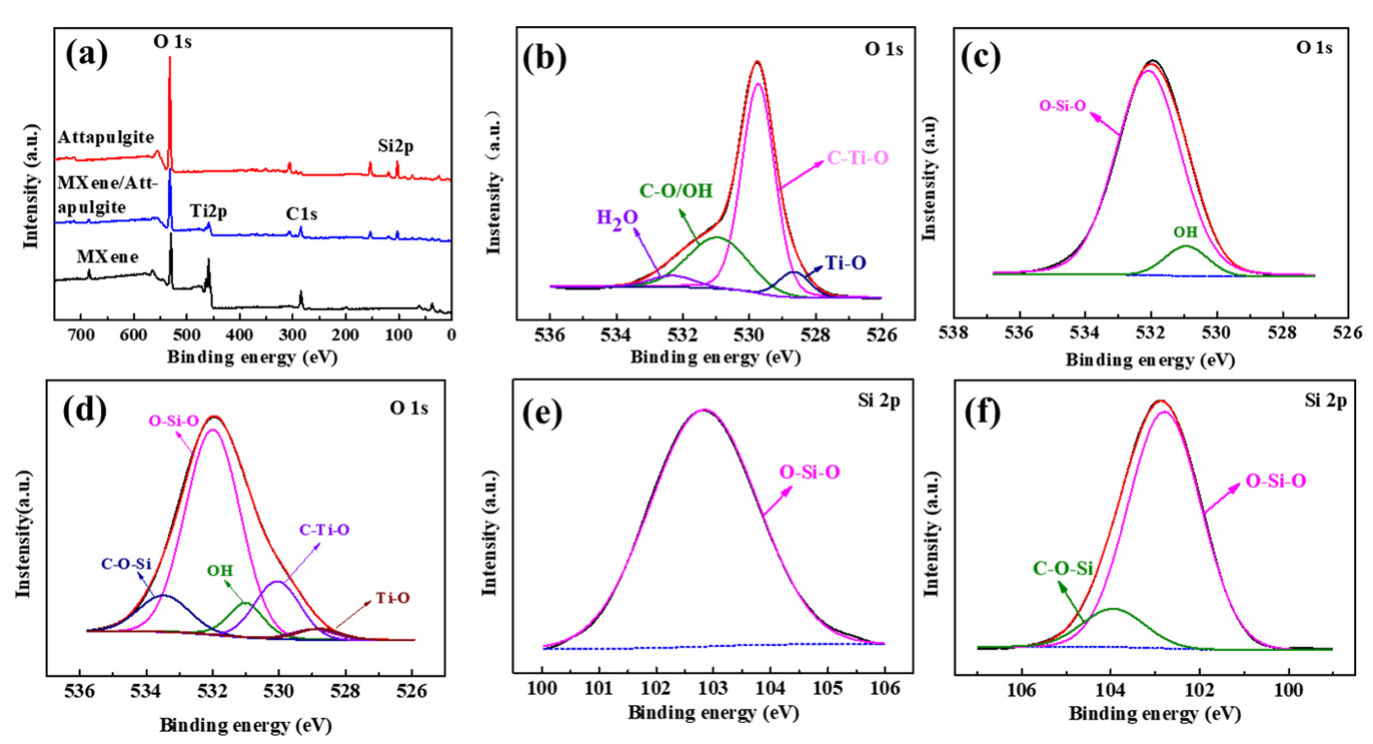
(**a**) XPS survey spectra of MXene, attapulgite, and MXene/attapulgite composite (1:2); high-resolution O 1s XPS spectra of (**b**) MXene, (**c**) attapulgite, and (**d**) MXene/attapulgite composite (1:2); high-resolution Si 2p XPS spectra of (**e**) attapulgite and (**f**) MXene/attapulgite composite (1:2).

The surface chemical states of MXene, attapulgite and the MXene/attapulgite composite (1:2) were studied by XPS (shown in Figure 3). Compared with MXene (Ti, O, C) and attapulgite (O, Si), the composite contains O, Ti, C, Si, which confirms the successful embedding of the attapulgite nanorods into the MXene nanosheets (shown in Figure 3a). The high-resolution O 1s spectrum of MXene (Figure 3b) can be fitted by four peaks (528.6, 529.7, 530.9 and 532.4 eV), which are assigned to Ti–O, C–Ti–O, C–O/OH and H_2_O, respectively, while only three peaks, identified as OH (530.9 eV) and Si–O–Si (532.1 eV), are observed in that of attapulgite (Figure 3c). As shown in Figure 3e, the characteristic peak located around 103.9 eV is assigned to O–Si–O in attapulgite [20]. All these characteristic peaks of O and Si elements in attapulgite or MXene exist simultaneously in the MXene/attapulgite composite (1:2) (Figure 3d,f). Additionally, there is a brand new peak that is noted here, which might be assigned to C–O–Si (533.4 eV of O 1s and 104.0 eV of Si 2p) and which verifies that the oxygen-containing functional groups of attapulgite and MXene reacted with each other to form the MXene/attapulgite composites [20]. The XPS results are in good agreement with the aforementioned FTIR analysis.

### 3.2. Morphological, Structural and Chemical Characterization of MXene/Attapulgite Composite Membranes

**Figure 4:**
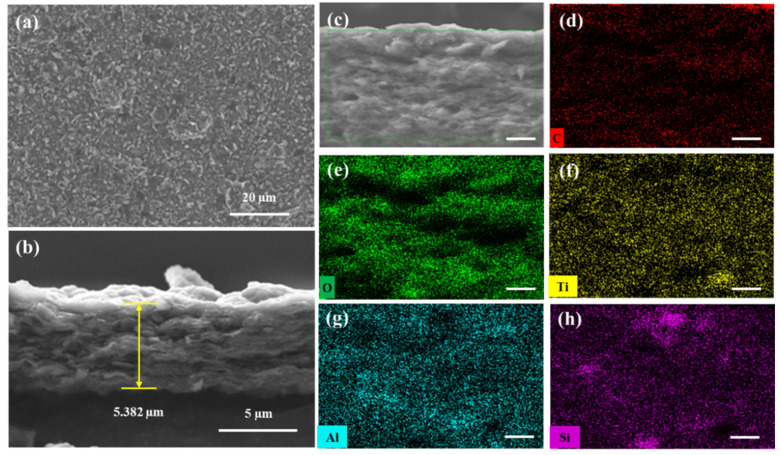
SEM images of the MXene/attapulgite (1:2) composite membrane: (**a**) top view and (**b**) cross-sectional view (magnified in (**c**)). Element distribution results of the MXene/attapulgite composite membrane (mapping from (**c**)) regarding different elements: (**d**) C, (**e**) O, (**f**) Ti, (**g**) Al, and (**h**) Si, scale bar: 1 μm.

The morphology of the MXene/attapulgite composite (1:2) was studied by SEM. With the incorporation of the attapulgite nanorods, the as-fabricated MXene/attapulgite membrane exhibits a rougher surface topography (Figure 4a) with a nacre-like lateral arrangement (Figure 4b). Its thickness is about 5.3 µm, which is about 2.2 µm thicker than that of pure MXene membrane, proving that attapulgite nanorods are inserted between the MXene nanosheets as pillars. On the other hand, the MXene interlayer spacing is increased to expand the internal two-dimensional channel for molecular transportation and to reduce the transmission resistance [29]. Therefore, this composite membrane is expected to present a high dye rejection with a rather limited water permeation flux loss. The distribution of elements across the cross-section of the composite membrane (Figure 4c) is investigated by energy dispersive spectroscopy (EDS) analysis that includes C, O, Ti, Al, and Si, as shown in Figure 4d–h. Besides C, O and Ti belonging to MXene, it is noted that Al and Si that originated from attapulgite are observed in the dark area unoccupied by C and O. Moreover, both Al and Si are as evenly distributed in the image as Ti, revealing that attapulgite is uniformly incorporated into the composite membrane. These results indicate that the strategy developed in this study can induce a good intercalation of attapulgite nanorods between the MXene nanoflakes.

**Figure 5:**
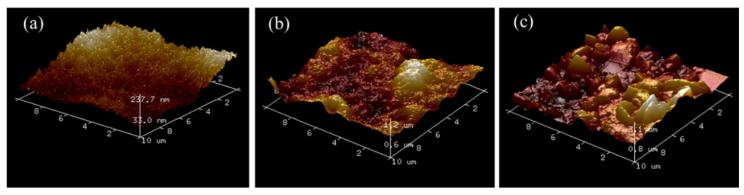
AFM diagrams of (**a**) pure MXene, (**b**) MXene/attapulgite composite (1:2), and (**c**) MXene/attapulgite composite (1:4).

The surface roughness of MXene and two MXene composite membranes were determined using AFM, as shown in Figure 5. It is obvious that the addition of attapulgite has a great impact on the surface roughness of the composite membranes. Compared with pure MXene membrane, the surface roughness of composite membranes increases with respect to attapulgite incorporation. This is attributed to the nano-size and rich oxygen-containing functional groups of attapulgite, which enable it to easily agglomerate and accumulate during the membrane fabrication process [30]. These results are also consistent with the SEM and EDS observations and may benefit dye molecules’ preferential retention on its surface.

### 3.3. Separation Performance of MXene/Attapulgite Composite Membranes

**Figure 6:**
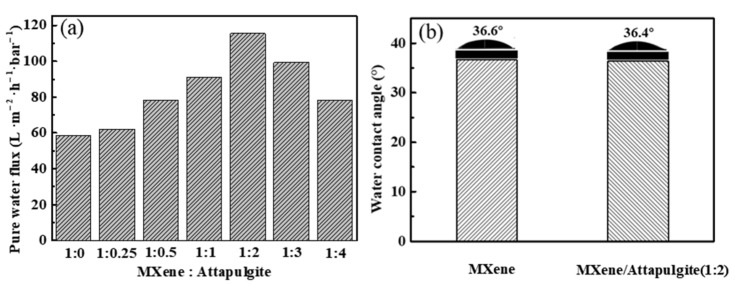
(**a**) Effect of mass ratio of MXene to attapulgite on the pure water flux of composite membranes; (**b**) water contact angle measurement of MXene and MXene/attapulgite composite (1:2) membrane.

The separation performance of MXene/attapulgite composite membranes was evaluated by their pure water permeation flux, surface hydrophilicity, and dye rejection rate. As shown in Figure 6a, the pure water flux of the MXene/attapulgite composite membranes increases with the increase of the attapulgite content all the way until the mass ratio of MXene to attapulgite reaches 1:2, where a maximum of 115.47 L·m^−2^·h^−1^·bar^−1^ is attained, which is 1.97 times that of MXene membrane. According to the Hagen-Poiseuille equation, this high pure water permeation flux results from the expanded interlayer spacing and possibly results in a better dye rejection [11]. Further increasing the attapulgite content leads instead to a decline in the flux, due to the thickness also being increased and to the elongated transmission channels, which account for a higher transmission resistance of the composite membranes [19]. In addition, the surface hydrophilicity of the MXene membrane and the MXene/attapulgite composite membrane (1:2) is studied by water contact angle measurement. Two similar water contact angle values are noted (36.6° and 36.4°, as shown in Figure 6b), indicating that both the MXene membrane and MXene/attapulgite composite membranes are highly hydrophilic. This finding also suggests that a uniform incorporation of attapulgite into the composite was realized here [19,31].

**Figure 7:**
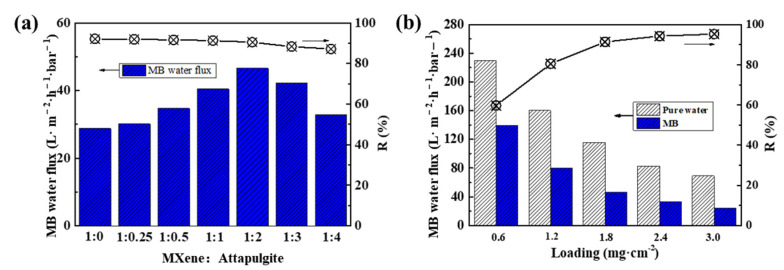
(**a**) Effect of mass ratio of MXene to attapulgite on MB flux and rejection rate of composite membranes; (**b**) effect of MXene/attapulgite composite loading on the obtained pure water flux, MB flux and rejection rate.

The effect of the attapulgite content on the MB solution flux and rejection rate of the composite membranes was carefully studied. As shown in Figure 7a, with an initial concentration of MB solution of 15 mg·L^−1^, the MB solution flux of MXene/attapulgite composite membrane increases with the attapulgite content until reaching a MXene/attapulgite ratio of 1:2 and then reverses, which is similar to that of the pure water flux. Meanwhile, the rejection rate for MB is relatively stable around 90% (90.67%). On the other hand, the highest permeation flux of MB is 46.51 L·m^−2^·h^−1^·bar^−1^, 61.72% higher than that of pure MXene membrane, though lower than for the pure water flux. It is speculated that during the process of dye rejection, MB molecules are increasingly intercepted onto the composite membranes and gradually block their interlayer transmission channels [32]. Furthermore, the basically unchanged rejection rate of the composite membranes implies that the MB rejection is not affected here by the used attapulgite content. Given that the suitable lateral size of pure MXene should mainly contribute to MB rejection, further intercalation of attapulgite into the composite membranes only has a limited negative effect. In other words, although attapulgite inserted between the MXene nanosheets can increase the interlayer spacing of the composite membranes, their internal nano-channels are still physically capable of rejecting MB that is within the specific molecular weight and concentration [33].

During the preparation process, the thickness of the composite membranes can be easily adjusted by altering the loading of the MXene/attapulgite suspension, which is one of the most important factors for a good dye rejection performance. As shown in Figure 7b, the water permeation fluxes of pure water and MB solution of MXene/attapulgite composite membranes decreased rapidly at a higher loading of MXene/attapulgite, which is contrary to the trend of the MB rejection rate. With the highest loading of 3.0 mg·cm^−2^, the MB flux can still reach 69.01 L·m^−2^·h^−1^·bar^−1^, with a rather high dye removal (95.29%).

## 4. Conclusions

In summary, we have demonstrated that the MXene/attapulgite composites can be readily loaded onto the microfiltration membrane by vacuum filtration, during which attapulgite is intercalated into the MXene membrane. Consequently, the nano-channel between interlayers was extended and was expected to increase the permeation flux effectively. Compared with pure MXene, the permeation fluxes of pure water and MB solution of MXene/attapulgite composite membrane are increased by 97.31% and 61.72%, respectively, while still maintaining a high MB rejection rate (90.67%). Further investigations indicated that the interlayer spacing of MXene nanosheets in the composites was rationally enlarged by the intercalated attapulgite nanorods, which enhanced the permeation flux without affecting the effective interception of MB. Overall, this study provides a new avenue for fast and efficient dye removal based on MXene/attapulgite composite membranes.

## Data Availability

Not applicable.

## References

[1] Naguib M., Kurtoglu M., Presser V., Lu J., Niu J., Min H., Hultman L., Gogotsi Y. (2011). Two-Dimensional Nanocrystals Produced by Exfoliation of Ti_3_AlC_2_. Adv. Mater..

[2] Wan S., Li X., Wang Y., Chen Y., Xie X., Yang R., Tomsia A., Jiang L., Cheng Q. (2020). Strong sequentially bridged MXene sheets. Proc. Natl. Acad. Sci. USA.

[3] Liu G., Shen J., Liu Q., Liu G., Xiong J., Yang J., Jin W. (2018). Ultrathin two-dimensional MXene membrane for pervaporation desalination. J. Membr. Sci..

[4] Ren C., Hatzell K., Alhabeb M., Zheng L., Gogotsi Y. (2015). Charge- and Size-Selective Ion Sieving through Ti_3_C_2_T_x_ MXene Membranes. J. Phys. Chem. Lett..

[5] Ihsanullah I. (2020). Potential of MXenes in Water Desalination: Current Status and Perspectives. Nano-Micro Lett..

[6] Lu Z., Wei Y., Deng J., Ding L., Wang H. (2019). Self-Crosslinked MXene (Ti_3_C_2_T_x_) Membranes with Good Antiswelling Property for Monovalent Metal Ion Exclusion. ACS Nano.

[7] Wei L., Deng W., Li S., Wu Z., Cai J., Luo J. (2022). Sandwich-like chitosan porous carbon Spheres/MXene composite with high specific capacitance and rate performance for supercapacitors. J. Bioresour. Bioprod..

[8] Gao Y., Wang L., Zhou A., Li Z., Chen J., Bala H., Hu Q., Cao X. (2015). Hydrothermal synthesis of TiO_2_/Ti_3_C_2_ nanocomposites with enhanced photocatalytic activity. Mater. Lett..

[9] Zhang S., Liao S., Qi F., Liu R., Xiao T., Hu J., Li K., Wang R., Min Y. (2020). Direct deposition of two-dimensional MXene nanosheets on commercially available filter for fast and efficient dye removal. Hazard Mater..

[10] Pandey R., Rasool K., Madhavan V., Aïssa B., Gogotsi Y., Mahmoud K. (2018). Ultrahigh-flux and fouling-resistant membranes based on layered silver/MXene (Ti_3_C_2_T_x_) nanosheets. J. Mater. Chem. A.

[11] Ding L., Wei Y., Wang Y., Chen H., Caro J., Wang H. (2017). A Two-Dimensional Lamellar Membrane: MXene Nanosheet Stacks. Angew. Chem. Int. Edit..

[12] Das R. (2021). Two-Dimensional (2D) Nanomaterial in Separation Science.

[13] Liu T., Liu X., Graham N., Yu W., Sun K. (2020). Two-dimensional MXene incorporated graphene oxide composite membrane with enhanced water purification performance. J. Membr. Sci..

[14] Long Q., Zhao S., Chen J., Zhang Z., Qi G., Liu Z. (2021). Self-assembly enabled nano-intercalation for stable high-performance MXene membranes. J. Membr. Sci..

[15] Huang R., Lin Q., Zhong Q., Zhang X., Wen X., Luo H. (2020). Removal of Cd(II) and Pb(II) from aqueous solution by modified attapulgite clay. Arab. J. Chem..

[16] Fan Z., Zhou S., Mao H., Li M., Xue A., Zhao Y., Xing W. (2021). A novel ceramic microfiltration membrane fabricated by anthurium andraeanum-like attapulgite nanofibers for high-efficiency oil-in-water emulsions separation. J. Membr. Sci..

[17] Dong S., Li B., Zhang J., Wang A. (2018). Superamphiphobic Coatings with Low Sliding Angles from Attapulgite/Carbon Composites. Adv. Mater. Interfaces.

[18] Yuan B., Yin X., Liu X., Li X., Sun L. (2016). Enhanced Hydrothermal Stability and Catalytic Performance of HKUST-1 by Incorporating Carboxyl-Functionalized Attapulgite. ACS Appl. Mater. Interfaces.

[19] Zhao X., Su Y., Liu Y., Jiang Z. (2016). Free-Standing Graphene Oxide-Palygorskite Nanohybrid Membrane for Oil/Water Separation. ACS Appl. Mater. Interfaces.

[20] Wang C., Zeng W., Jiang T., Chen X., Zhang X. (2019). Incorporating attapulgite nanorods into graphene oxide nanofiltration membranes for efficient dyes wastewater treatment. Sep. Purif. Technol..

[21] Naguib M., Mashtalir O., Carle J., Presser V., Lu J., Hultman L., Gogotsi Y., Barsoum M. (2012). Two-Dimensional Transition Metal Carbides. ACS Nano.

[22] Shi J., Li M. (2020). Surface modification effects in phase change material-infiltrated attapulgite. Mater. Chem. Phys..

[23] Zhang X., Tian J., Wang P., Liu T., Ahmad M., Zhang T., Guo J., Xiao H., Song J. (2022). Highly-efficient nitrogen self-doped biochar for versatile dyes’ removal prepared from soybean cake via a simple dualtemplating approach and associated thermodynamics. J. Clean. Prod..

[24] Li J., Xu C., Zhang Y., Wang R., Zha F., Shea H. (2016). Robust superhydrophobic attapulgite coated polyurethane sponge for efficient immiscible oil/water mixture and emulsion separation. J. Mater. Chem. A.

[25] Dong S., Li S., Li Z., Zhang L., Yin L. (2018). Mesoporous Hollow Sb/ZnS@ C Core–Shell Heterostructures as Anodes for High-Performance Sodium-Ion Batteries. Small.

[26] Xing R., Pan F., Zhao J., Cao K., Gao C., Yang S., Liu G., Wu H., Jiang Z. (2016). Enhancing the permeation selectivity of sodium alginate membrane by incorporating attapulgite nanorods for ethanol dehydration. RSC Adv..

[27] Li M., Zhou S., Xue A., Su T., Zhang Y., Zhao Y., Xing W. (2015). Fabrication of porous attapulgite hollow fiber membranes for liquid filtration. Mater. Lett..

[28] Chang C., Nie X., Li X., Tao P., Fu B., Wang Z., Xu J., Ye Q., Zhang J., Song C. (2020). Bioinspired roll-to-roll solar-thermal energy harvesting within form-stable flexible composite phase change materials. J. Mater. Chem. A.

[29] Wang X., Sun M., Wang R., Jiao L., Bian H., Dai H. (2022). Promoting h-BN Dispersion in Cellulose-based Composite by Lignosulfonate for Regulatable Effectual Thermal Management. Mater. Des..

[30] Fu D., Wang R., Wang Y., Sun Q., Cheng C., Guo X., Yang R. (2022). An easily processable silver nanowires-dual-cellulose conductive paper for versatile flexible pressure sensors. Carbohyd. Polym..

[31] Wang Y., Ou R., Wang H., Xu T. (2015). Graphene oxide modified graphitic carbon nitride as a modifier for thin film composite forward osmosis membrane. J. Membr. Sci..

[32] Fan X., Cai C., Gao J., Han X., Li J. (2020). Hydrothermal reduced graphene oxide membranes for dyes removing. Sep. Purif. Technol..

[33] Wang R., Yang R., Zhang Y. (2017). A study of applying green glucose-reduced graphene oxide in advanced treatment of different dyes. Desalin. Water Treat..

